# Inhibition of Browning in Apples Using Betacyclodextrin-Assisted Extracts of Green Rooibos (*Aspalathus linearis*)

**DOI:** 10.3390/foods12030602

**Published:** 2023-02-01

**Authors:** Lusani Norah Vhangani, Jessy Van Wyk

**Affiliations:** Department of Food Science and Technology, Cape Peninsula University of Technology, Bellville 7535, South Africa

**Keywords:** green rooibos, encapsulation, betacyclodextrin, polyphenols, hydroxymethyl furfural, non-enzymatic browning

## Abstract

Green rooibos’ bioactive compounds contribute greatly towards its antioxidant activity. The anti-browning activity of aqueous (GRE) and beta-cyclodextrin (β-GRE)-assisted extracts of green rooibos was investigated in canned apples. Freeze-dried extracts (GRE and β-GRE) obtained at 40 °C for 60 min were added in canned apples at 0.25 and 0.5% prior to heat processing and stored at 23 and 37 °C for 24 weeks. Lightness (L*), colour difference (DE*), furfural and hydroxymethyl furfural (HMF) were determined to establish the effect of extracts against non-enzymatic browning (NEB) development. The L* value decreased, whereas DE*, HMF and furfural increased with increased storage time and temperature. A higher inhibition was observed for samples stored at 23 °C, and storage at 37 °C reduced (*p* < 0.05) the inhibitory capacity of extracts. Greater inhibition against NEB development was reported for β-GRE 0.25 and 0.5 via the L* value (40.93–46.67%), β-GRE 0.25 for DE* (46.67%) and β-GRE 0.25 and 0.5 for HMF (59.55–67.33%). No differences (*p* > 0.05) were observed in furfural inhibition between all extracts, although inhibition was reported at 62.69–72.29%. Browning inhibition correlated with the reaction rate constant (k_0_) and activation energy (Ea), exhibiting a correlation coefficient of 0.925, 0.964, 0.932 and 0.754 for L*, DE*, HMF and furfural, respectively.

## 1. Introduction

**Figure 1:**
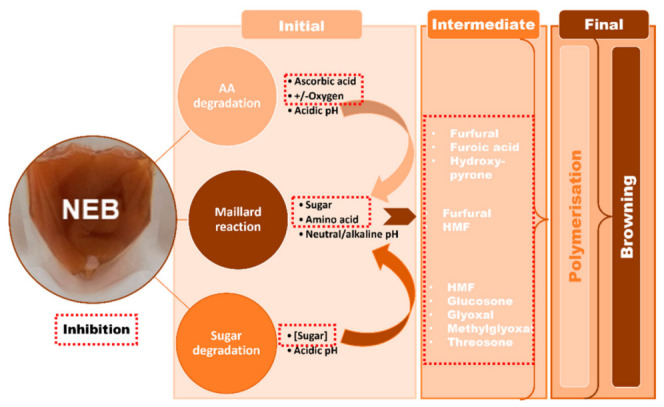
Non-enzymatic browning mechanisms and inhibition in fruit and fruit-based products.

Non-enzymatic browning (NEB) reactions in fruit and fruit-based products have been attributed to ascorbic acid (AA) and sugar degradation pathways. In the case of apple and its derivatives, the Maillard reaction (MR) and sugar degradation have been reported as the primary cause of browning during the processing and storage of apple juice [1,2,3,4] and puree [5] due to the high sugar and low AA contents (Figure 1). However, AA degradation was reported in apple puree supplemented with 0.5 and 1 g.kg^−1^ vitamin C [6,7]. Browning renders food products undesirable to consumers and may even lead to the formation of toxic compounds [8]. In light of this information, preventing NEB reactions is of utmost importance.

Chemical inhibitors are the most preferred choice due to their low cost and high performance. Amongst them, sulphites have proven to be superior in blocking the initial condensation step of the MR via attaching to the carbonyl of sugars (Figure 1) or stabilising the intermediate hydroxymethyl furfural (HMF) [9]. Another synthetic advanced glycation end product (AGE) inhibitor, aminoguanidine (AG), is known as an excellent trapper of methylglyoxal (MGO) [10]. However, these inhibitors have been implicated in safety concerns [10,11,12].

Plant extracts are alternative candidates due to their versatile constituents with bioactivities. Crude plant extract bioactives have been assessed for their ability to inhibit NEB markers such as HMF, furfural, glyoxal (GO), methyl glyoxal (MGO) and the development of advance glycation end products (AGEs) [10,13,14,15]. Using model systems of bovine serum albumin (BSA) and sugar or sugar only, Wu et al. [14] showed that guava leaf and fruit extracts exhibited a slight inhibitory effect against the formation of Amadori products and good inhibitory activity against dicarbonyl compounds and AGEs. Moreover, its activity was higher than that of AG. The inhibitory capacity was attributed to the presence of polyphenolic compounds, namely, gallic acid, catechin, quercetin and ferulic acid, whose anti-glycation properties were linked to their antioxidant activity [12,15,16,17].

Of interest in this current study, several authors reported on the phenolic content of green rooibos. The flavonoids and phenolic acids class are the major phenolic compounds in green rooibos [17,18,19,20]. Based on the results reported by the authors above, aspalathin and nothofagin are the main flavonoids, with others such as quercetin present at lower concentrations. Moreover, Joubert and DeBeer [21] further reported that aspalathin was a potent antioxidant compared favourably with epigallocatechin gallate (EGCG). Moreover, gallic and ferulic acid mentioned above were amongst other phenolic acids reported in green rooibos.

Although plant extracts or polyphenols thereof have been proven to exhibit excellent anti-browning activities in vitro, the final intended application is in food processing and storage. Crude plant extracts have been reported to contribute their colour and flavour when incorporated into food. This is influenced by either natural pigments, flavours or the reaction of polyphenols with other food components resulting in polymerisation [17]. These changes are also accelerated by the high temperatures employed during heat processing, leading to reduced bioactivities. For instance, 1–1.5% EGCG successfully reduced the browning rate of bread rolls; however, at increased concentrations of 3%, it exhibited pro-oxidative activity [22]. These authors suggested that darkening occurred due to the autoxidation of polyphenols at high temperatures. These limitations may pose a challenge using native plant extracts [23].

The encapsulation of sensitive compounds provides improved stability during processing, masks off flavours or odours and prevents reactions with other components in food products, such as oxygen [24]. Common encapsulation material includes carbohydrates, protein and lipid polymers such as maltodextrin, inulin [25], soy protein isolates, sodium alginate and cyclodextrins, to mention a few. The choice of polymer is crucial, as it affects how the active compound is released. For instance, Hidalgo et al. [26] reported elevated furosine levels in soy protein isolates applied to encapsulate beetroot pomace due to thermal treatments. Similarly, maltodextrin reacts with glycine to form browning compounds [27]. Lavelli and Sri Harsha [28] reported that sodium alginate-encapsulated grape skin extracts exhibited a reduced potential to inhibit glycation of fructose-BSA and MGO-BSA model systems; this was due to some phenolics interacting with alginate, resulting in a reduced bioactivity. Therefore, cyclodextrins were the obvious choice, since they were proven not to participate in any NEB reactions [27]. Moreover, unlike other potential encapsulants, beta-cyclodextrin (β-CD) is also known to aid in the extraction of these polyphenols from their sources, hence the term “β-CD-assisted extracts” [29,30]. Maraulo et al. [30] proved that β-CD enhanced the physical properties of olive pomace extracts via improved heat stability, reduced hygroscopicity, and increased antioxidant activity. Regarding NEB inhibition using β-CD-encapsulated plant extracts, Favre et al. [29,31] proved that β-CD-assisted extracts of thyme and green pepper were effective in retarding browning development (A_420nm_) and HMF formation in glucose-BSA model systems, respectively.

Therefore, this study aimed to apply β-CD-assisted green rooibos extracts in canned apples during processing and storage to inhibit NEB reactions.

## 2. Materials and Methods

### 2.1. Green Rooibos and Reagents

The dry green rooibos was obtained from a major local producer (Rooibos Ltd., Clanwilliam, South Africa). Beta-cyclodextrin (β-CD) was purchased from Industrial Analytical (Kyalami, South Africa). Golden Delicious apples were purchased from a local supermarket (Bellville, South Africa). Apple concentrate (70 °B) was purchased from Associated Fruit Processors (Paarl, South Africa). Hydroxymethyl furfural, ascorbic acid, thiobarbituric acid, Carrez I and II, trichloroacetic acid, fructose, glucose, sucrose, methanol and sulphuric acid were purchased from Merck (Modderfontein, South Africa). Furfural was purchased at Sigma-Aldrich (Kempton Park, South Africa). The chemicals used in this study were of analytical grade, and chemical reagents were prepared according to standard analytical procedures. Prepared reagents were stored under conditions that prevented deterioration or contamination. The water used was purified with the Milli-Q water purification system (Millipore, Microsep, Bellville, South Africa).

### 2.2. Solid–Liquid Extraction of Green Rooibos and Analysis of Bioactive Compounds

Green rooibos, as received, was coarsely milled (Fritsch, Idar-Oberstein, Germany) using a sieve with an aperture of 0.2 mm. The extraction of green rooibos was performed based on the method of El Darra et al. [32] and Cai et al. [33] with slight modifications. Green rooibos of 10 g and 100 mL of 0 and 15 mM β-CD (1.7%) aqueous solutions (1:10 (*w*/*v*)) in a Schott bottle were homogenised using a Polytron homogeniser at 29,000 rpm for 2 min, followed by heating the mixture at 40 °C on a temperature-controlled heating mantle for 60 min with magnetic stirring at 1500 rpm. The extracts were cooled immediately and centrifuged at 10,000 rpm for 15 min at 4 °C. The supernatant was freeze-dried, and the resulting powder, termed native/aqueous green rooibos extracts (GRE) and β-CD-assisted extracts of green rooibos (β-GRE), were stored in air-tight containers at −20 °C until further analysis. The bioactive compounds in GRE and β-GRE were analysed using ACQUITY ultra-performance liquid chromatography (UPLC) coupled with a photodiode array (PDA) detector and a SYNAPT G_2_S high-definition mass spectrometer (HDMS).

### 2.3. The Canning Process

The preparation and process of canning was conducted following the guidelines stipulated by the South African Agricultural Product Standards Act (APSA) 119 of 1990 [34]. Apple concentrate of 70 °Brix (°B) was diluted to a 30 °B, followed by the addition of 0.25 and 0.5% green rooibos extracts (15 mM β-CD: 40 °C:60 min) as prepared in Section 2.2. To evaluate the effect of β-CD encapsulation, aqueous green rooibos extracts (0 mM β-CD: 40 °C: 60 min) were also prepared. Apples were peeled, cored and dipped in an ice-cold 2% solution of calcium chloride [35], after which they were sliced longitudinally from the calyx to the stem into segments of not less than 40 mm in height with thicknesses ranging from 16–20 mm [34]. Apple slices (70 g) were weighed into retortable laminated polyethylene terephthalate (PET) polyethylene (PE) ethylene vinyl alcohol (EVOH) of 160 mm × 100 mm self-standing pouches. Ice-cold juice concentrate (30 °B) was filled to a final mass of 120 g, and the pouches were sealed immediately with a pedal heat sealer. The sealed pouches were heat-processed in a horizontal retort at 100 °C for 20 min. Temperature profiles in the retort and at the coldest point of the sample were recorded using type T-thermocouples (Ellab, Hillerod, Denmark). Canned apples were stored in temperature-controlled incubators at 23 and 37 °C. Initial sampling was executed prior to storage (i.e., immediately after retorting); after that, sampling was performed monthly. At each sampling period, canned pouches were taken randomly from the incubators.

### 2.4. Sample Preparation

During specific sampling days, the temperature of canned samples was equilibrated in ice-cold water. Samples were then homogenised into a puree using a stick blender (Mellerware, Cape Town, South Africa). The pureed samples were used as they are for the measurement of Brix (°B), pH and colour. All the other analyses were performed using water extracts of the puree. Two grams of apple puree were diluted with 4 mL Milli-Q water, vortexed for 1 min, and then centrifuged (Beckman Coulter, Brea, CA, USA) at 12,000× *g* for 10 min. A 1 mL aliquot of the supernatant was mixed with 50 μL of Carrez I and 50 μL of Carrez II solutions, allowed to stand for 30 min, followed by centrifugation at 10,000 rpm for 5 min [4].

### 2.5. Browning Indices Colour Measurement (L, a*, b*)

The colour of the pureed apple samples was evaluated by measuring the CIELab parameters L* (brightness, 100 = white, 0 = black), a* (+red; −green) and b* (+yellow; −blue) utilising a spectrophotometer (CM-5, Konika Minolta, Tokyo, Japan), measuring the colour spectra using a D65 daylight source, a large viewing area and the observer at 10°. The colour difference was calculated using the following formula:

(1) $${\mathrm{DE}}^{*}=\sqrt{{\left({\Delta L}^{*}\right)}^{2}+{\left({\Delta a}^{*}\right)}^{2}+{\left({\Delta b}^{*}\right)}^{2}}$$

where:

ΔL* is the difference in lightness/darkness value.

Δa* is the difference in red/green.

Δb* is the difference in yellow/blue.

The percentage inhibition of browning was calculated following the formula described by [9].

% Browning inhibition L* = [(ΔL*_control_ − ΔL*_sample_)/(ΔL*_control_)] × 100 (2)

where:

ΔL*_control_ is the difference between the measured L* value for the control at week 24 and the corresponding value at week 0.

ΔL*_sample_ is the difference between the measured L* value for the sample with added extract at week 24 and the corresponding value at week 0.

% Browning inhibition ΔDE* = [(ΔDE*_control_ − ΔDE*_sample_)/(ΔDE*_control_)] × 100 (3)

where:

ΔDE*_control_ is the difference between the calculated DE* for the control at week 24 and the corresponding value at week 4.

ΔDE*_sample_ is the difference between the measured DE* for the sample with added extract at week 24 and the corresponding value at week 4.

### 2.6. Determination of 5-hydroxymethyl Furfural and Furfural

Furfural and 5-hydroxymethyl furfural contents were determined according to the spectrophotometric method described by Bharate and Bharate [9] and Liao et al. [36], with slight modifications. Each 2 mL aliquot of sample was mixed with 2 mL of 12% trichloroacetic acid and 2 mL of 0.025 M thiobarbituric acid, reacted at 40 ± 0.5 °C for 50 min and cooled to room temperature after the reaction. The absorbance was measured at 436 nm for furfural and 443 nm for HMF. The HMF and furfural concentrations were evaluated through calibration standard curves capturing the range from 0 to 6.6 mg.L^−1^. The A436 nm and A443 nm were measured to determine the content of furfural and 5-HMF, and this was calculated based on regression equations from standard curves with an average R^2^ of 0.9996 and 0.9998 for furfural and HMF, respectively. The furfural and HMF values obtained for each sample for the 24 weeks were then used to calculate the percentage inhibition. The percentage of furfural and HMF inhibition was calculated following the formulae:

% Furfural inhibition = [(ΔFurfural_control_ − ΔFurfural_sample_)/(ΔFurfural_control_)] × 100 (4)

where:

ΔFurfural_control_ is the difference between the furfural value for the control at week 24 and the corresponding value at week 0.

ΔFurfural_sample_ is the difference between the furfural value for the sample with added extract at week 24 and the corresponding value at week 0.

% HMF inhibition = [(ΔHMF_control_ − ΔHMF_sample_)/(ΔHMF_control_)] × 100 (5)

where:

ΔHMF_control_ is the difference between the HMF value for the control at week 24 and the corresponding value at week 0.

ΔHMF_sample_ is the difference between the HMF value for the sample with added extract at week 24 and the corresponding value at week 0.

### 2.7. Statistical Analysis

The inhibitory effect of GRE and β-GRE in canned apples against NEB reactions was determined via percentage inhibition of lightness (L*), the colour difference (DE*) and the formation of intermediate products (HMF and furfural) calculated following Equations (2)–(5). In addition, zero-order reaction kinetics Equations (6)–(8) for each index were applied to determine the effect of added extracts in reducing the reaction rate constant (k_0_) as well as the regression coefficient (R^2^) thereof. The effect of temperature on *k* was calculated from the Arrhenius equation (Equation (10)) to determine the activation energy (Ea), or Equation (11).

Statistical analysis was performed using SPSS 27.0 for Windows^®^. Analysis of variance (ANOVA) established the significance of each dependent factor. Descriptive statistical analyses determined the triplicates’ mean and standard deviation (*n* = 3). Duncan’s multiple range tests determined significant differences among means. The level of confidence required for significance was selected at 95%.

(6) $$L*={L*}_{0}+k_{0}t$$

(7) $$\Delta \mathrm{DE}*={\Delta \mathrm{DE}*}_{0}+k_{0}t$$

(8) $$\mathrm{HMF}={\mathrm{HMF}}_{0}+k_{0}t$$

(9) $$\mathrm{Furfural}={\mathrm{Furfural}}_{0}+k_{0}t$$

(10) $$\ln k=\ln k_{0}-\frac{\mathrm{Ea}}{\mathrm{RT}}$$

(11) Ea=−RT⋅lnkA

where L*, DE*, HMF and Furfural is the concentration at time t; L*_0_, ΔDE*_0_, HMF_0_ and Furfural_0_ is the concentration at time zero; *k*_0_ is the zero-order rate constant; and t is the storage time. T is the absolute temperature in °K, Ea is the activation energy, and R is the universal gas constant (8.3145 J.mol^−1^ K^−1^).

## 3. Results and Discussion

### 3.1. Inhibition of Colour Formation Via L* Value and DE*

The results of colour development via the L* values and DE* of canned apples with added green rooibos extracts (GRE and β-GRE) stored at 23 and 37 °C are shown (Supplementary Figure S1, Tables S1 and S2). Initially, all samples displayed varying levels of L* values. As expected, the control exhibited the highest (*p* < 0.05) L* value of 58.91 at week zero (Table S1). Upon the addition of green rooibos extracts (GRE and β-GRE), the red component (a*) increased (results not shown), and brightness (L*) decreased (*p* < 0.05) due to GRE and β-GRE imparting the natural red colour to the canned samples, albeit no significant differences (*p* > 0.05) were observed amongst the L* values of the samples initially (Table S1). This change was also visible to the naked eye and agrees with the observation that crude plant extracts or purified individual polyphenols affect colour changes when incorporated into food or model systems (Figure S1). This colour change can be ascribed to either natural pigmentation or the reaction of polyphenols with food components resulting in colour augmentation [25,36,37].

For instance, Hidalgo et al. [26] experienced colour change when they added native and encapsulated beetroot pomace extract (BPE) during water biscuit baking. They observed a sharp decrease (*p* < 0.05) in L* and an increase in a* values due to the presence of betacyanins. In the present study, at the end of storage, the control and GRE reported the lowest L* value (34.58–37.42) at 23 °C, and at 37 °C, it was observed for β-GRE and GRE at 0.5%. Similarly, Hidalgo et al. [26] also reported a greater reduction in L* values as the BPE concentration increased from 14.1–5. They observed a sharp decrease (*p* < 0.05) in L* and an increase in a* values due to the presence of betacyanins. In the present study, at the end of storage, the control and GRE reported the lowest L* value (34.58–37.42) at 23 °C, (Table S1), and at 37 °C, it was observed for β-GRE and GRE at 0.5%. Similarly, Hidalgo et al. [26] also reported a more significant reduction in L* values as the BPE concentration increased from 5.7–14.1%.

Based on these results, it is evident that the decrease (*p* < 0.05) in the L* value was more pronounced at 37 °C compared to 23 °C, and this is attributed to the influence of temperature on browning development [38]. Furthermore, the brown colour development of samples was further explained via DE*, where an increase (*p* < 0.05) was observed during storage (Table S2). Therefore, reporting on the changes in the colour of each sample cannot be directly compared to the control, even more so because there are variations in the extract concentrations. Hence, it was worth determining the percentage (%) inhibition of browning via colour formation for each concentration, as well as the reaction rate constant (k_0_) and activation energy Ea, with the latter two being more accurate in determining the progression of the reaction and inhibitory effect of added extracts.

The % inhibition was used to describe the effectiveness of added GRE and β-GRE in reducing the decrease in lightness (L*) during storage, i.e., in inhibiting browning. Table 1 depicts the % inhibition of colour formation via the L* value and DE*. The %IL* value ranged from 0.20–46.67%, with the lowest value reported for GRE 0.5 (0.2%) and β-GRE 0.5 (2.44) at 37 °C, and the highest for β-GRE 0.5 (46.67%) at 23 °C. The inhibitory effect of β-GRE increased as the extract concentration increased. On the other hand, no significant differences (*p* > 0.05) were observed as a function of concentration for GRE samples stored at 23 °C. However, as the storage temperature increased to 37 °C, the overall %IL* value was reduced, and it worsened (*p* < 0.05) as the concentration increased to 0.5% for both extract types. Moreover, an opposite trend was observed at 23 °C, where GRE 0.25 samples exhibited a higher % inhibition capacity than β-GRE 0.25. Percentage (%) inhibition is a good indicator of the inhibitory effect of extracts; however, it must be noted that it only considers the initial and final L* values, without reflecting the rest of the storage weeks. Therefore, the determination of the rate constant (k) becomes a suitable method to supplement the % inhibition.

Hence, to further confirm the effect of the addition of green rooibos extracts against the browning of canned apples, kinetics was applied to establish the reaction rate constant (k) for each temperature for the duration of storage. Table 1 depicts the rate constant of zero-order (k_0_) reactions for samples with added extracts. At all storage temperatures, GRE and β-GRE markedly decreased (*p* < 0.05) the browning rate relative to the control; as expected, the highest (*p* < 0.05) k_0_ values were reported for the control at 0.9786 and 1.1130 compared to GRE and β-GRE. Favreau-Farhadi et al. [22] studied the inhibition kinetics of epigallocatechin gallate (EGCG) and rosmarinic acid (RA) against the browning of applesauce and bread rolls. They, too, reported a higher rate constant (k_1_) for the control compared with 1–1.5% EGCG and 1% RA-added samples. At 23 °C, the k_0_ decreased (*p* < 0.05) in the order of control > GRE 0.5 = GRE 0.25 = β-GRE 0.25 > β-GRE 0.5. However, at 37 °C, the order of decrease in k_0_ was control > β-GRE 0.5 > GRE 0.25 = GRE 0.55 = β-GRE 0.25. This implies that amongst the samples with added green rooibos extract, the rate of colour formation at 37 °C progressed faster in β-GRE 0.5 compared to all samples. Numerous authors have reported the anti-browning properties of crude plant extracts or purified bioactives thereof, where polyphenols were linked to antioxidant activity. In the present study, the individual bioactive compounds content of β-GRE was mostly higher than that of GRE, particularly aspalathin, as shown in Table S3. The present paper is a small segment of a bigger study. In contrast, in our previous paper [39], the antioxidant activity of GRE was reported, particularly the superior activity of β-CD-assisted extracts (β-GRE) exhibited via metal chelation and radical scavenging. Moreover, these were correlated to the total polyphenolic content, with aspalathin content being the highest amongst the determined flavonoids [39]. The results leading to colour development and reduction of the rate thereof were associated with the action of polyphenols in scavenging radicals produced during reducing sugar dehydration, which leads to the formation of dicarbonyl compounds and eventually browning via polymerisation. However, the reduction in inhibitory capacity, in particular, β-GRE 0.5, can be attributed to increased concentration and release of polyphenols, which in the presence of heat result in autooxidation, resulting in browning, and not necessarily the Maillard reaction, AA or sugar dehydration-associated browning. Another argument can be that the natural red pigment in green rooibos polymerised. Favreau-Farhadi et al. [22] added EGCG (3%) and RA (1.5%) in bread roll dough and readymade applesauce. They found that the rate of browning in the bread rolls accelerated with increased concentrations of EGCG (3%), and RA (1.5%) and was higher (*p* < 0.05) than that of the control. Unlike in the present study, these polyphenols did not initially impact any colour in the dough. However, high temperatures employed during baking had an influence, since browning was not experienced in the applesauce. It is worth noting that the applesauce was readymade and then stored at 37 °C for 30 days. In the present study, GRE and β-GRE were incorporated in canned apples before retorting, followed by storage for 24 weeks at 37 °C. Therefore, for applesauce, the temperature of 37 °C was low compared to temperatures employed during baking. Hence, the autoxidation of polyphenols was not initiated in the applesauce, but in the bread rolls. Favreau-Farhadi et al. [22] reported that polyphenols have optimal concentrations and/or conditions as antioxidants, beyond which they promote oxidation, and this phenomenon is evident in the results of the present study, in which high concentrations of polyphenols in β-GRE and high retorting temperatures resulted in increased browning in apples.

Colour difference describes colour variations by considering lightness (L*), a* (red/green) and b* (yellow/blue) coordinates [40]. Therefore, DE* is a more accurate predictor of colour formation than the individual L*a*b* coordinates and is a direct reflection of noticeable changes that can be perceived visually. As mentioned in the previous section, a DE* of 1 is the threshold at which a trained observer would notice the colour difference between two objects. In contrast, a DE* between 4 and 8 is deemed acceptable, but above 8, DE* is deemed unacceptable, and the product is likely to be rejected by consumers [41]. The colour difference of the canned apples with added extracts ranged from 1.66 to 31.02, as depicted in Table S2. In the present study, samples stored at 23 °C were still deemed acceptable at week 8, after which DE* drastically increased beyond unacceptable ranges. However, at 37 °C, only GRE 0.25 could reach eight weeks within the acceptable range. All the other samples with added extracts maintained the acceptable threshold until four weeks of storage.

The percentage inhibition of DE* is displayed in Table 1. At 23 °C, an increase in the concentration of extracts resulted in an increase (*p* < 0.05) in %IDE*, and this increase was more significant (*p* < 0.05) for β-GRE compared to GRE-added samples. Meanwhile, at 37 °C, no significant differences (*p* > 0.05) in %IDE* were observed between GRE 0.25, 0.5 and β-GRE 0.25 extracts, and a further drastic decrease was observed for β-GRE 0.5 at −17%. This correlates with the results reported for the L* value regarding loss of the inhibitory effect by β-GRE 0.5. Although the L* value for β-GRE 0.5 was comparable to that of GRE 0.5, in terms of % IDE*, β-GRE 0.5 exhibited a negative result, which usually denotes pro-oxidative properties due to autooxidation. Qi et al. [37] found that flavan-3-ols added at higher concentrations of 0.3–1 mg.L^−1^ in fried potatoes to mitigate NEB reactions also resulted in a significant increase (*p* < 0.05) in DE*, which was not experienced at 0.01–0.1%. It is worth mentioning that the highest %IDE* was reported for β-GRE 0.5% at 23 °C, which is similar to that which was reported for the %IL* value. This is expected, since DE* is derived from the L* value, while considering the contribution of the a* and b* coordinates.

Moreover, the k_0_ for DE*, as presented in Table 1, reported similar results to that which was obtained for L* value. The progression of colour change at 23 °C for the control was equal (*p* > 0.05) to that of GRE 0.25 and 0.5, with all β-GRE samples proving to significantly (*p* < 0.05) reduce the rate of colour change more than the control and all GRE samples. Furthermore, at 37 °C, the control, β-GRE 0.5 and GRE 0.25 exhibited a similar k_0_, with only GRE 0.5 and β-GRE 0.25 showing inhibitory activity towards reducing the rate of colour change. Looking at colour development holistically, at the lower temperature of 23 °C, β-GRE exhibited the highest %IL* value and %IΔE. However, at a higher storage temperature of 37 °C, the rate of browning intensified, resulting in a reduction in the inhibitory capacity of β-GRE, with the lower capacity being more pronounced in β-GRE 0.5 than in β-GRE 0.25. Concerning GRE samples, at a concentration of 0.25%, these extracts showed no changes (*p* > 0.05) in inhibitory capacity for both the L* value and DE*, whereas the inhibitory capacity of GRE 0.5 decreased. Both native and encapsulated green rooibos extracts effectively reduced overall browning in canned apples at 23 °C, an ambient temperature at which canned food products are likely to keep throughout their shelf-life. This then proves that these may be suitable anti-browning agents. However, sensory evaluation studies have to be conducted to evaluate the effect of green rooibos addition in terms of taste, flavour and overall acceptability.

### 3.2. Inhibition of Furfural and HMF Formation

The furfural content of canned apples with added green rooibos extracts (GRE and β-GRE) stored at 23 and 37 °C is depicted in Table S4. The furfural content ranged from 1.42–24.92 mg. 100 g^−1^, with no significant differences (*p* > 0.05) observed between the control and all samples at week zero. The highest furfural content was reported as 4.18 and 24.92 mg.100 g^−1^ for control samples at 23 and 37 °C at week 24. No significant increases (*p* > 0.05) were observed in the furfural content of GRE and β-GRE stored at 23 °C. This implies the inhibitory effect of these extracts at 23 °C. However, significant increases (*p* < 0.05) were observed at 37 °C as the storage time increased, indicating the effect of increased temperature on accelerating furfural formation and the inhibitory effect of each sample type.

To evaluate the impact of added extracts on furfural formation during storage, the inhibition capacity was calculated based on the control concerning each sample with the added extract. The results depicted in Table 2 reveal that GRE and β-GRE exhibited excellent inhibition capacity at 23 °C against furfural formation at 62.69–72.29%, even though no significant differences were observed between them.

As stated previously, ascorbic acid becomes oxidised to dehydroascorbic acid in the presence of oxygen. The possible inhibition mechanism of furfural formation was due to the donation of hydrogen to reduce dehydroascorbic acid back to ascorbic acid. That way, ascorbic acid degradation, and consequently furfural formation, are delayed. As the storage temperature increased to 37 °C, a significant reduction in furfural inhibition was observed, with the lowest amount reported at 10.17% for GRE 0.25. At higher temperatures, NEB reactions are favoured, such as sugar hydrolysis followed by dehydration to form highly unstable radicals. Thus, GRE and β-GRE’s inhibitory effect diminishes, since there are more reactions to combat. Therefore, greater inhibition in furfural formation was observed for samples stored at 23 vs. 37 °C.

Likewise, as described by reaction rate constants, furfural formation showed that the control sample at 23 °C exhibited a significantly higher k_0_ (*p* < 0.05) compared to GRE and β-GRE treated samples. Moreover, the lack of a significant difference (*p* > 0.05) observed between all samples with added extracts is in accordance with that which was reported in % furfural inhibition at the same temperature (Table 2). At 37 °C, the reaction rates were, on average, 12 times higher than those reported at 23 °C, again highlighting the effect of elevated temperature on increased reaction rates, as has been observed repeatedly. No significant differences (*p* < 0.05) were observed between the control sample and GRE 0.25%. Meanwhile, the k_0_ for GRE 0.5, β-GRE 0.25 and 0.5 was significantly lower (*p* < 0.05) than for the control sample, but not different from each other (*p* < 0.05). The inhibition of furfural formation by various polyphenols has been reported in the literature, even though the focus was more on model systems than real food. Oral et al. [42] found polyphenols (tyrosol, epicatechin, punicalagin, chlorogenic, caffeic and ellagic acid) and extracts from olive mill waste, pomegranate peel and European cranberry juice were effective against inhibiting furfural formation in glycine-glucose model systems produced at higher temperatures of 140 and 180 °C. The inhibitory effect of GRE and β-GRE is linked to their antioxidant activity, particularly the radical scavenging by donating hydroxyl groups to trap reactive carbonyl species produced during ascorbic acid degradation as glucose and fructose autoxidation. The radical scavenging activity of GRE and β-GRE was reported in the present study of Vhangani et al. [39].

Regarding HMF, the content ranged between 0.61–5.67 mg.100 g^−1^ (Table S5). Similar to furfural, the highest HMF content was reported for the control sample after 24 weeks of storage at 37 °C. Overall, the HMF content was lower than furfural, which may be attributed to the sugar composition of the canned apples. The canned apples were comprised of 66% fructose, 22% glucose and 12% sucrose of the total sugar (results not shown). Based on the composition, it was speculated that furfural formation was achieved via the oxidative degradation of ascorbic acid, sugar dehydration and the Maillard reaction, with fructose being the main precursor rather than glucose. On the other hand, sucrose was hydrolysed to yield its invert sugars (results not included). For this reason, the furfural content was higher than HMF.

In terms of the inhibitory effect of green rooibos extracts, the % HMF inhibition increased significantly (*p* < 0.05) as the concentration of GRE increased from 0.25 to 0.5%; meanwhile, no significant differences (*p* > 0.05) were observed between β-GRE samples at both storage temperatures, although the highest inhibitions were recorded for β-GRE at 59.55 to 67.33% (Table 2). At 37 °C, a similar trend was observed, whereby an increase in GRE concentration resulted in increased % inhibition of HMF, with no significant difference observed for β-GRE. However, it should be noted that an overall decrease in inhibition was reported for all extracts as a function of increased storage temperature. To confirm the inhibitory action in the aforementioned results, the k_0_ values of the control sample were significantly higher (*p* < 0.05) than that of all samples with varying concentrations of extracts for both 23 and 37 °C. The rate of HMF formation was reduced by the addition of increasing concentrations of GRE. The lowest k_0_ was reported for both β-GRE at 0.25 and 0.5, since no significant differences were observed between them. Similarly, Favre et al. [29] also correlated total phenolic contents and antioxidant activities, with the antiglycation properties of green, white and black peppers via HMF inhibition.

The study of Qi et al. [37] mentioned earlier proved the link between anti-browning capacity and inhibitory activity against HMF formation. Flavan-3-ols at 50, 100 and 150 µg.mL^−1^ showed a dose-dependent inhibition against HMF formation in glucose-asparagine model systems. Meanwhile, in a real food system, the reduction of HMF formation in fried potato chips dipped in 0.01–0.1 mg.mL^−1^ of flavan-3-ols also increased with increased concentration; however, concentrations higher than 0.1 mg.mL^−1^ caused a decline in inhibition. This phenomenon also coincided with the reduction in lightness and increase in DE*. High concentrations of added phenols that resulted in the darkening of the chips also resulted in reduced HMF reduction. Therefore, as the polyphenols participate in autoxidation, their inhibitory effect against NEB is also reduced. In the present study, we also reported the decline in the inhibitory effect of GRE and β-GRE against HMF.

To conclude on the holistic inhibitory effect of GRE and β-GRE, each extract’s activation energy (Ea) for each dependent variable is shown in Table 3. The activation energy (Ea) is defined as the minimum energy required to start a chemical reaction. In this instance, the effect of temperature is combined for 23 and 37 °C for each sample treatment over the storage period calculated from the Arrhenius equation [38,43]. In terms of browning development via the L* value and DE*, the control and all GRE samples exhibited the lowest Ea (*p* < 0.05), revealing that GRE was a less effective inhibitor against browning development. Although the Ea of GRE 0.25 for the L* value was not significantly different from β-GRE 0.25 (*p* > 0.05), β-GRE 0.5 showed the highest Ea of 36.33 kJ.mol^−1^, indicating greater inhibitory power via resisting the initiation of browning.

Concerning intermediate NEB product formation, no significant differences were observed (*p* > 0.05) between the Ea of HMF for the control and GRE 0.25 samples at 49.12 and 47.23 kJ.mol^−1^, respectively. The Ea of GRE 0.5 and β-GRE 0.5 was comparable, which was equal to that of β-GRE 0.25. Regarding furfural, the highest Ea of 167.13 kJ.mol^−1^ was reported for GRE 0.25, followed by β-GRE 0.25 at 148.82 kJ.mol^−1^, and no significant differences were observed between GRE 0.5 and β-GRE 0.5 (*p* > 0.05). Overall, the samples treated with green rooibos extracts exhibited the highest Ea compared to the control. Therefore, furfural formation required less energy to form in the control samples and was restricted by the presence of GRE and β-GRE.

## 4. Conclusions

The results of this study revealed that green rooibos extracts could inhibit browning development, with β-GRE 0.25 and 0.5 exhibiting a higher ability to reduce browning development. However, as the temperature increased to 37 °C, the inhibitory effect of green rooibos was reduced. The formation of HMF and furfural followed the same trend. When comparing the β-GRE, 0.25 and 0.5 exhibited a higher inhibition of HMF compared to GRE, although no significant differences (*p* > 0.05) were observed between furfural inhibition. It is worth noting that furfural inhibition ranged from 62–72%. These results were further proved via activation energy (Ea), whereby the control sample exhibited Ea comparable to that of GRE for brown development and HMF, and β-GRE exhibited a high Ea, which indicated resistance to browning and furfural formation.

## Figures and Tables

**Table 1:** Inhibitory capacity of green rooibos extracts against browning of canned apples during storage.

Sample Type		Lightness Value (L*)			Colour Difference (DE*)		
Conc []	Temp ( °C)	% IL*	(k_0_)	r^2^	% IDE*	(k_0_)	r^2^
Control	23	-	−0.9786 ± 0.38 ^d^	0.9286	-	1.0205 ± 0.69 ^b^	0.9377
Gre 0.25	23	18.45 ± 3.64 ^c^	−0.7536 ± 0.39 ^bc^	0.9575	8.94± 0.99 ^b^	0.8984 ± 1.09 ^b^	0.9582
Gre 0.5	23	17.07 ± 1.93 ^c^	−0.8283 ± 0.17 ^c^	0.9454	21.23 ± 1.20 ^c^	0.8555± 1.91 ^b^	0.9546
β-Gre 0.25	23	40.93 ± 2.71 ^d^	−0.6620 ± 0.55 ^b^	0.6918	36.22 ± 4.33 ^d^	0.6551 ± 1.32 ^a^	0.6803
β-Gre 0.5	23	46.67 ± 5.96 ^e^	−0.5325 ± 0.81 ^a^	0.8946	45.24 ± 3.06 ^e^	0.6106 ± 0.23 ^a^	0.8957
Control	37	-	−1.1130 ±1.27 ^C^	0.9670	-	1.0785± 0.51 ^C^	0.9465
Gre 0.25	37	18.55 ± 2.44 ^c^	−0.9199 ± 1.42 ^A^	0.9730	9.68 ± 1.46 ^b^	0.9954 ± 1.00 ^BC^	0.9321
Gre 0.5	37	0.20 ± 0.98 ^a^	−0.9179 ± 1.83 ^A^	0.9705	9.54 ± 1.3 ^b^	0.9262 ± 2.04 ^AB^	0.9582
β-Gre 0.25	37	9.82 ± 1.68 ^b^	−0.9210 ± 0.43 ^A^	0.9208	11.96 ± 2.15 ^b^	0.8657 ± 1.42 ^A^	0.9060
β-Gre 0.5	37	2.83 ± 0.83 ^a^	−1.0361± 0.744 ^B^	0.9622	−17.17 ± 6.51 ^a^	1.0929 ± 0.47 ^C^	0.9454

Data presented as % inhibition of decrease in lightness (% IL*) and increase in colour difference (% IDE*), reaction rate constant k_0_ (week ^−1^) and regression coefficient (r^2^) of colour change in canned apples with added green rooibos extracts stored at 23 and 37 °C for 24 weeks expressed as mean ± standard deviation (*n* = 3). Gre 0.25–green rooibos native extract at 0.25%, β-GRE 0.25–beta-cyclodextrin encapsulated green rooibos extract. ^a–e,A–C^ Means with different letter superscripts of the same case (low/upper) on the same column denote significant differences (*p* < 0.05).

**Table 2:** Inhibitory capacity of green rooibos extracts against the formation of furfural and HMF in canned apples during storage.

Sample Type		Furfural			HMF		
Conc []	Temp ( °C)	%IF	(k_0_)	r^2^	%Inhibition	(k_0_)	r^2^
Control	23	-	0.1241 ± 0.55 ^b^	0.7787	-	0.0865 ± 0.85 ^d^	0.9511
Gre 0.25	23	62.69 ± 1.46 ^c^	0.0320 ± 0.69 ^a^	0.7081	12.75 ± 12.75 ^a^	0.0746 ± 0.14 ^c^	0.9297
Gre 0.5	23	64.55 ± 4.16 ^c^	0.0379 ± 0.24 ^a^	0.8948	50.20 ± 3.56 ^d^	0.0459 ± 0.60 ^b^	0.9567
β-Gre 0.25	23	72.29 ± 1.20 ^c^	0.0322 ± 0.12 ^a^	0.8688	59.55 ± 5.64 ^e^	0.0273 ± 0.46 ^a^	0.8002
β-Gre 0.5	23	63.54 ± 9.57 ^c^	0.0376 ± 0.17 ^a^	0.8676	67.33 ± 5.22 ^e^	0.0301 ± 0.47 ^a^	0.9121
Control	37	-	0.8657± 1.42 ^B^	0.9388	-	0.2124 ± 0.08 ^D^	0.9751
Gre 0.25	37	10.17 ± 1.34 ^a^	0.7160 ± 1.6 ^AB^	0.8802	11.51 ± 1.21 ^a^	0.1774 ± 0.08 ^C^	0.8998
Gre 0.5	37	47.10 ± 1.51 ^b^	0.4892 ± 0.47 ^A^	0.9414	24.03 ± 8.39 ^b^	0.1573 ± 0.11 ^B^	0.9274
β-Gre 0.25	37	35.04 ± 3.09 ^b^	0.5275 ± 0.77 ^A^	0.8763	36.56 ± 3.14 ^c^	0.1215 ± 0.04 ^A^	0.8797
β-Gre 0.5	37	43.87 ± 1.51 ^b^	0.4877 ± 0.69 ^A^	0.9485	45.77 ± 2.62 ^cd^	0.1143 ± 0.61 ^A^	0.9518

Data presented as % inhibition of furfural and hydroxymethyl furfural in canned apples with added green rooibos extracts stored at 23 and 37 °C for 24 weeks expressed as mean ± standard deviation (*n* = 3). Gre 0.25–green rooibos native extract at 0.25%, β-GRE 0.25–beta-cyclodextrin encapsulated green rooibos extract. ^a–e,A–D^ Means with different letter superscripts on the same column denote significant differences (*p* < 0.05).

**Table 3:** Activation energy (kJ.mol^−1^) of green rooibos extracts against browning of canned apples during storage.

	Browning Development		Intermediate Products	
Sample type	L* Value	DE*	HMF	Furfural
Control	7.03 ± 1.79 ^a^	3.16 ± 3.33 ^a^	49.12 ± 7.12 ^a^	101.88 ± 3.53 ^a^
Gre 0.25	10.83 ± 2.15 ^ab^	5.87 ± 4.70 ^a^	47.23 ± 3.53 ^a^	167.13 ± 5.34 ^d^
Gre 0.5	5.55 ± 1.72 ^a^	4.42 ± 2.31 ^a^	67.42 ± 3.10 ^b^	140.06 ± 1.83 ^b^
β-Gre 0.25	18.84 ± 10.28 ^b^	15.54 ± 3.64 ^b^	81.92 ± 5.64 ^c^	148.82 ± 3.54 ^c^
β-Gre 0.5	36.33 ± 1.39 ^c^	32.06 ± 7.47 ^c^	73.12 ± 8.54 ^bc^	136.79 ± 3.63 ^b^

Data presented as activation energy KJ.mol^−1^ for lightness (L*-value), the colour difference (DE*), hydroxymethyl furfural (HMF) and furfural of canned apples with added green rooibos extracts stored at 23 and 37 °C for 24 weeks expressed as mean (*n* = 3). Gre 0.25–green rooibos native extract at 0.25%, β-GRE 0.25–beta-cyclodextrin encapsulated green rooibos extract. ^a–d^ Means with different letter superscripts on the same column denote significant differences (*p* < 0.05).

## Data Availability

Data from this project/study are stored in the Cape Peninsula University of Technology institutional repository and will be available on request.

## Supplementary Materials

The following supporting information can be downloaded at: https://www.mdpi.com/article/10.3390/foods12030602/s1: Figure S1: Canned apples with added GRE and β-GRE stored for week 0 (a), week 4 (b), week 12 (c) and week 24 (d). Table S1: Lightness (L*) of canned apples with added crude green rooibos extracts. Table S2: Colour difference (DE*) of canned apples with added green rooibos extracts. Table S3: Mass spectral data obtained from green rooibos extracts. Table S4: Furfural content of canned apples with added green rooibos extracts. Table S5: Hydroxymethyl furfural content of canned apples with added green rooibos extracts.
