# Supplementary material for: Inhibition of Browning in Apples Using Betacyclodextrin-Assisted Extracts of Green Rooibos (Aspalathus linearis)
Source: Foods. 2023 Feb 1;12(3):602. doi: 10.3390/foods12030602 (PMC9914553; doi:10.3390/foods12030602)
Supplement: Supplementary file 1 [file foods-12-00602-s001.zip › foods-2088540-supplementary.pdf]

Figure S1: Canned apples with added GRE and β-GRE stored for week 0 (a), week 4 (b), week 12 (c), and week 24 (d)

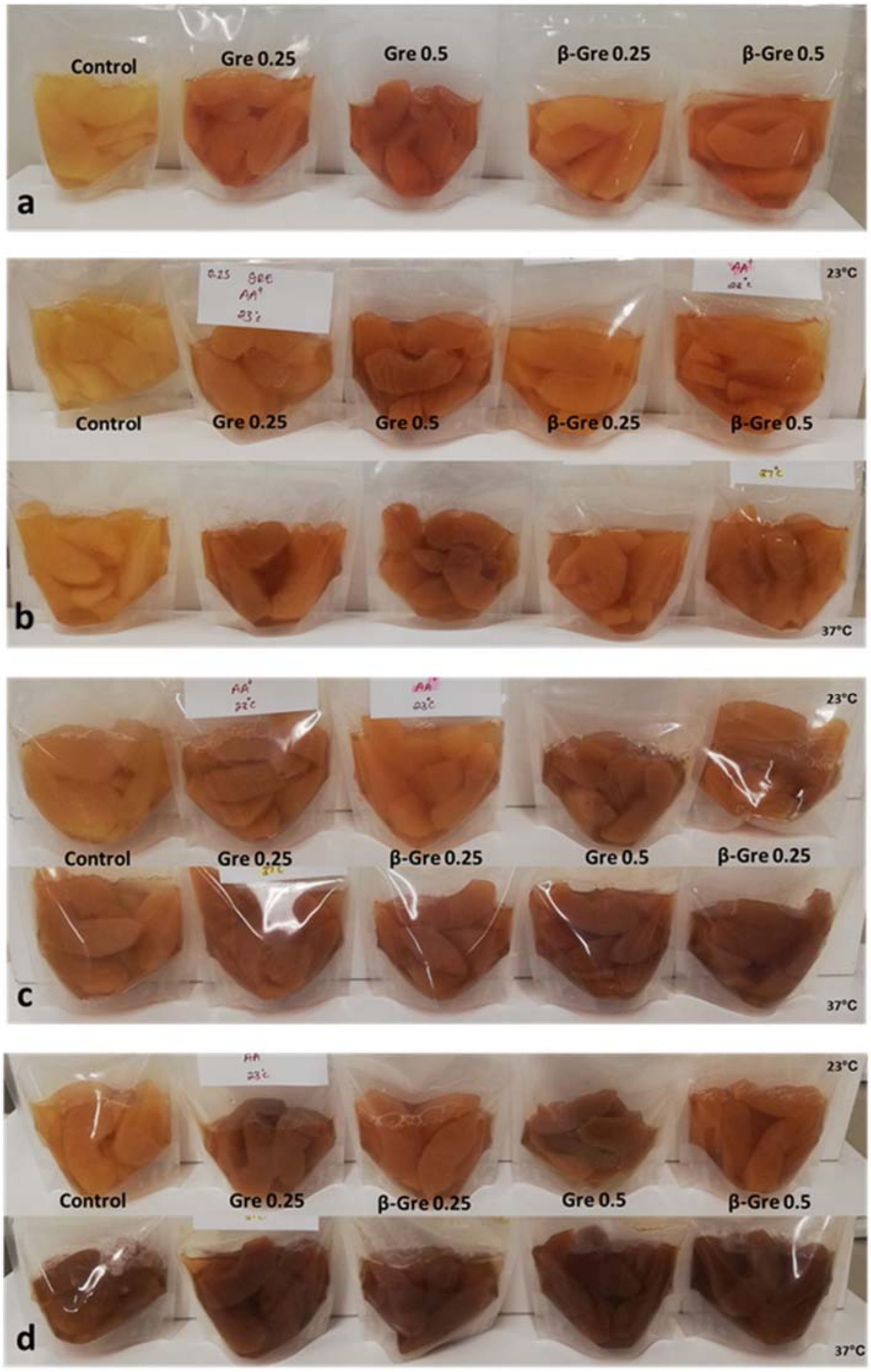

Table S1: Lightness (L*) of canned apples with added crude green rooibos extracts.

Storage		Samples type and concentration				
**Temp**	**Time**	**Control**	**Gre 0.25**	**Gre 0.5**	**β-Gre 0.25**	**β-Gre 0. 5**
23°C	0	58.91 ± 1.92^{k}	53.80 ± 1.75^{ij}	52.39 ± 1.87^{hij}	54.15 ± 0.99^{j}	52.55 ± 0.61^{hij}
	4	57.6 ± 1.50^{k}	52.15 ± 0.45^{hij}	50.72 ± 0.81^{hi}	53.69 ± 0.53^{hij}	50.73 ± 0.80^{hi}
	8	57.45 ± 2.14^{k}	50.85 ± 1.72^{hij}	50.38 ± 2.37^{h}	52.91 ± 0.58^{hij}	51.06 ± 2.09^{hij}
	12	46.55 ± 2.05^{g}	43.71 ± 1.20^{defg}	44.22 ± 2.08^{efg}	40.04 ± 1.03^{bc}	44.35 ± 1.56^{efg}
	16	45.89 ± 2.10^{fg}	42.94 ± 1.17^{cdef}	41.30 ± 0.89^{cde}	40.75 ± 2.40^{cd}	44.3 ± 0.88^{efg}
	20	41.48 ± 0.88^{cde}	40.17 ± 1.53^{bc}	35.59 ± 1.57^{a}	41.53 ± 1.91^{cde}	41.48 ± 1.89^{cde}
	24	37.42 ± 1.11^{ab}	36.29 ± 1.70^{a}	34.58 ± 1.31^{a}	41.54 ± 4.87^{cde}	41.09 ± 0.66^{cde}
37°C	0	58.91 ± 1.92^{R}	53.8 ± 1.75^{PQ}	52.39 ± 1.87^{PQ}	54.15 ± 0.99^{Q}	52.55 ± 0.61^{PQ}
	4	53.45 ± 2.05^{PQ}	50.86 ± 2.56^{OP}	43.75 ± 2.66^{KL}	48.51 ± 0.82^{NO}	51.06 ± 1.04^{OPQ}
	8	48.05 ± 0.96^{NO}	46.13 ± 0.91^{MN}	41.84 ± 3.87^{IJK}	39.9 ± 1.16^{HIJK}	42.93 ± 0.64^{KL}
	12	41.54 ± 0.79^{IJK}	42.14 ± 0.97^{JKL}	39.22 ± 1.53^{GHI}	39.6 ± 1.87^{GHIJ}	37.81 ± 0.70^{FGH}
	16	38.2 ± 1.12^{FGH}	38.71 ± 3.13^{GHI}	36.33 ± 2.25^{EFG}	35.31 ± 1.20^{DEF}	36.59 ± 0.77^{EFG}
	20	34.69 ± 0.74^{DE}	34.03 ± 1.79^{CDE}	32.66 ± 0.87^{CD}	33.79 ± 0.89^{CDE}	33.92 ± 0.36^{CDE}
	24	33.14 ± 1.69^{CD}	33.16 ± 2.95^{CD}	26.67 ± 3.88^{A}	31.11 ± 0.19^{BC}	27.41 ± 0.50^{A}

Data presented as Lightness (L* value) of canned apples with added green rooibos extracts stored at 23 and 37°C for 24 weeks expressed as mean ± standard deviation (n = 3). Gre 0.25 – green rooibos native extract at 0.25%, β-GRE 0.25 – betacyclodextrin encapsulated green rooibos extract.

Table S2: Colour difference (ΔE*) of canned apples with added crude green rooibos extracts.

Storage		Samples type and concentration				
**Temp**	**Time**	**Control**	**Gre 0.25**	**Gre 0.5**	**β-Gre 0.25**	**β-Gre 0. 5**
23°C	4	3.72 ± 1.10^{ab}	1.66 ±1.13^{a}	4.01 ± 1.24^{ab}	3.06 ± 0.56^{ab}	1.89 ± 0.91^{a}
	8	4.25 ± 1.03^{ab}	4.31 ± 0.05^{ab}	4.87 ± 1.53^{b}	4.36 ± 1.03^{ab}	1.67 ± 1.07^{a}
	12	13.46 ± 2.40^{fi}	11.27 ± 1.57^{df}	10.30 ± 1.76^{ce}	15.39 ± 2.56^{hi}	8.40 ± 0.94^{c}
	16	14.15 ± 1.02^{gi}	12.94 ± 2.63^{eh}	12.82 ± 1.14^{eh}	14.76 ± 1.01^{gi}	9.22 ± 1.26^{cd}
	20	19.07 ± 1.81^{k}	15.99 ± 1.36^{ij}	18.30 ± 1.28^{jk}	14.56 ± 1.08^{gi}	12.04 ± 1.18^{eh}
	24	23.27 ± 2.69^{l}	19.46 ± 1.36^{k}	19.41 ± 1.36^{k}	15.52 ± 1.64^{hi}	12.61 ± 0.18^{eg}
37°C	4	10.00 ± 1.79^{BD}	2.93 ±1.28^{A}	6.85 ±1.66^{B}	6.99 ± 1.20^{B}	2.15 ±1.03^{A}
	8	15.19 ± 2.04^{FG}	7.34 ± 1.68^{B}	9.01 ± 1.48^{BC}	14.88 ± 1.80^{FG}	8.92 ± 1.01^{BC}
	12	21.02 ± 2.68^{IK}	11.49 ± 1.37^{CE}	12.59 ± 1.72^{DF}	15.65 ± 1.37^{FG}	14.35 ± 1.82^{EG}
	16	24.61 ± 0.76^{KL}	15.90 ± 1.69^{F}	15.83 ± 1.45^{G}	20.35 ± 2.02^{IJ}	16.29 ± 1.13^{GH}
	20	29.28 ± 2.50^{M}	20.65 ± 2.95^{IJ}	19.55 ± 0.99I	22.87 ± 1.59^{JL}	19.08 ± 1.57^{H}
	24	31.02 ± 1.69^{M}	21.94 ± 2.64^{IK}	25.83 ± 1.14^{L}	25.49 ± 2.41^{L}	26.78 ± 1.73^{L}

Data presented as colour difference (ΔE*) of canned apples with added green rooibos extracts stored at 23 and 37°C for 24 weeks expressed as mean ± standard deviation (n = 3). ^{abc} Means with different letter superscripts of the same upper or lowercase on the same row denotes significant differences ($p < 0.05$) GRE 0.25 – green rooibos native extract at 0.25%, β-GRE 0.25 – betacyclodextrin encapsulated green rooibos extract.

Table S3: Mass spectral data obtained from green rooibos extracts.

Bioactive characteristics					Green rooibos content (mg.kg^{-1})	
**Identified compound**	**Ontology**	**Molecular Formula**	**RT**	**m/z**	**GRE**	**β-GRE**
4-O-Methylgalactinol	O-glycosyl compounds	$C_{13}H_{24}O_{11}$	5.19	355.12	47.29^a	88.59^b
Cellulose, microcrystalline	O-glycosyl compounds	$C_{14}H_{26}O_{11}$	6.28	369.14	159.71^a	244.57^b
Xanthohumol B	2'-Hydroxychalcones	$C_{21}H_{22}O_6$	6.40	369.14	154.57^a	222.12^b
MINEs-59993	Shikimic acids and derivatves	$C_8H_{10}O_7$	6.66	217.03	65.12^a	84.22^a
1-O-Caffeoylglucose	Hydroxycinnamic acid glycosides	$C_{15}H_{18}O_9$	7.23	341.09	273.26^a	345.46^b
4-Fumarylacetoacetic acid	Medium-chain keto acids and derivatives	$C_8H_8O_6$	7.56	199.02	61.88^a	83.75^a
2,3-Butanediol apiosylglucoside	O-glycosyl compounds	$C_{15}H_{28}O_{11}$	7.74	383.16	215.66^a	285.53^b
gentesic acid 5-O-glucoside	Phenolic glycosides	$C_{13}H_{16}O_9$	7.85	315.07	205.30^a	291.99^b
(2R,3S)-Piscidic acid	Phenylpropanoic acids	$C_{11}H_{12}O_7$	8.01	255.05	656.66^a	869.20^b
Kanzonol N	5-O-methylated isoflavonoids	$C_{22}H_{24}O_6$	8.30	383.15	187.84^a	270.07^b
Pseudolaroside B;(-)-Pseudolaroside B	Hydrolyzable tannins	$C_{14}H_{18}O_9$	8.44	329.09	117.38^a	124.92^a
7-hydroxy-4-{3-oxo-3H-benzo[f]chromen-2-yl}-2H-chromen-2-one	Naphthopyrans	$C_{22}H_{12}O_5$	8.68	355.06	277.06^a	430.26^b
5,7-Dihydroxyflavone 7-benzoate	Flavones	$C_{22}H_{14}O_5$	8.86	357.08	283.00^a	467.38^b

Identified compound	Ontology	Molecular Formula	RT	m/z	GRE	β-GRE
Chlorogenic acid	Quinic acids and derivatives	$C_{16}H_{18}O_9$	9.48	353.09	223.55[a]	336.05[b]
Diethyl tartrate	Beta hydroxy acids and derivatives	$C_8H_{14}O_6$	9.64	205.07	619.10[a]	864.16[b]
3'-Methoxyfukiic acid	Phenylpropanoic acids	$C_{12}H_{14}O_8$	9.95	285.06	179.47[a]	241.84[b]
1-O-Galloylglycerol	Galloyl esters	$C_{10}H_{12}O_7$	10.04	243.05	337.02[a]	468.54[b]
1-O-Caffeoylglucose	Hydroxycinnamic acid glycosides	$C_{15}H_{18}O_9$	10.52	341.09	447.64[a]	648.29[b]
UNPD25930	Saccharolipids	$C_{17}H_{30}O_{12}$	10.82	425.17	44.00[a]	71.40[b]
2'-(E)-Feruloyl-3-(arabinosylxylose)	Coumaric acids and derivatives	$C_{20}H_{26}O_{12}$	11.12	457.14	100.21[a]	137.38[a]
3-O-p-Coumaroylquinic acid	Quinic acids and derivatives	$C_{16}H_{18}O_8$	11.18	337.09	266.47[a]	394.51[b]
4',6'-Dihydroxy-2'-methoxyacetophenone 6'-glucoside	Phenolic glycosides	$C_{15}H_{20}O_9$	11.31	343.10	83.37[a]	113.87[a]
Epicatechin	Catechins	$C_{15}H_{14}O_6$	11.48	289.07	125.76[a]	213.45[b]
Homovanillic acid	Methoxyphenols	$C_9H_{10}O_4$	11.66	181.05	119.57[a]	184.17[b]
4-Hydroxycinnamic acid	Hydroxycinnamic acids	$C_9H_8O_3$	11.82	163.04	95.07[a]	194.79[b]
(1S,6R)-2-succinyl-6-hydroxycyclohexa-2,4-diene-1-carboxylic acid	Gamma-keto acids and derivatives	$C_{11}H_{12}O_6$	11.94	239.06	170.10[a]	220.49[b]
Phenyl pyruvic acid-2-O-β-D-glucoside related	Hydroxycinnamic acid glycosides	$C_{15}H_{18}O_8$	12.11	325.09	715.45[a]	1114.67[b]

Identified compound	Ontology	Molecular Formula	RT	m/z	GRE	β-GRE
UNPD90386	Phenolic glycosides	$C_{21}H_{26}O_{13}$	12.42	485.13	100.69[a]	163.45[b]
2'-(E)-Feruloyl-3-(arabinosylxylose)	Coumaric acids and derivatives	$C_{20}H_{26}O_{12}$	12.74	457.14	163.34[a]	281.01[a]
Paeonoside	Phenolic glycosides	$C_{15}H_{20}O_{8}$	13.13	327.11	338.81[a]	576.05[b]
UNPD221285	Xanthones	$C_{26}H_{30}O_{16}$	13.34	597.15	580.04[a]	945.01[b]
Quercetin 3-galactoside-7-glucoside	Flavonoid-7-O-glycosides	$C_{27}H_{30}O_{17}$	13.69	625.14	243.85[a]	352.93[b]
(S)-eriodictyol-6-C-b-D-glucopyranoside	Flavonoid-3-O-glycosides	$C_{21}H_{22}O_{11}$	13.93	449.11	293.24[a]	437.26[b]
Xeroboside	Coumarin glycosides	$C_{21}H_{26}O_{13}$	14.03	485.13	211.03[a]	345.78[b]
Carlinoside		$C_{26}H_{28}O_{5}$	14.16	579.13	82.72[a]	161.45[b]
Okanin 4'-alpha-L-arabinofuranosyl-(1->4)-glucoside	Flavonoid O-glycosides	$C_{26}H_{30}O_{15}$	14.45	581.15	341.50[a]	701.23[b]
(R)-eriodictyol-6-C-b-D-glucopyranoside	Flavonoid-3-O-glycosides	$C_{21}H_{22}O_{11}$	14.48	449.10	159.99[a]	291.21[b]
Catechin 3',5-diglucoside	Flavonoid O-glycosides	$C_{27}H_{34}O_{16}$	14.76	613.18	200.50[a]	425.58[b]
(S)-eriodictyol-8-C-b-D-glucopyranoside	Flavonoid-3-O-glycosides	$C_{21}H_{22}O_{11}$	15.00	449.11	113.41[a]	159.67[b]
UNPD88203	Flavonoid-3-O-glycosides	$C_{26}H_{32}O_{16}$	15.07	599.16	489.80[a]	981.61[b]
(R)-eriodictyol-8-C-b-D-glucopyranoside	Flavonoid-3-O-glycosides	$C_{21}H_{22}O_{11}$	15.20	449.11	125.19[a]	218.86[b]

Identified compound	Ontology	Molecular Formula	RT	m/z	GRE	β-GRE
Phenyl pyruvic acid-2-O-β-D-glucoside	Hydroxycinnamic acid glycosides	$C_{15}H_{18}O_8$	15.45	325.09	1428.66[a]	2368.87[b]
Catechin 3',5-diglucoside	Flavonoid O-glycosides	$C_{27}H_{34}O_{16}$	15.64	613.18	143.64[a]	249.94[b]
Apiin	Flavonoid-7-O-glycosides	$C_{26}H_{28}O_{14}$	15.71	563.14	111.96[a]	215.08[b]
Orientin	Flavonoid-3-O-glycosides	$C_{21}H_{20}O_{11}$	15.88	447.09	278.11[a]	582.10[b]
Catechin 3',4'-diglucoside	Flavonoid O-glycosides	$C_{27}H_{34}O_{16}$	16.14	613.18	369.18[a]	663.32[b]
Aspalathin	2'-Hydroxy-dihydrochalcones	$C_{21}H_{24}O_{11}$	16.45	451.12	729.11[a]	1496.20[b]
Quercetin robinobioside	Flavonoid-3-O-glycosides	$C_{27}H_{30}O_{16}$	17.24	609.15	326.51[a]	621.32[b]
Vitexin	Flavonoid-O-glycosides	$C_{21}H_{20}O_{10}$	17.37	431.09	237.26[a]	438.10[b]
Rutin	Flavonoid-3-O-glycosides	$C_{27}H_{30}O_{16}$	17.40	609.14	378.09[a]	785.35[b]
Quercetin hexoside_1	Flavonoid-3-O-glycosides	$C_{21}H_{20}O_{12}$	17.70	463.09	252.28[a]	491.04[b]
Isovitexin	Flavonoid-O-glycosides	$C_{21}H_{20}O_{10}$	17.76	431.09	245.44[a]	463.44[b]
Quercetin hexoside_2	Flavonoid-3-O-glycosides	$C_{21}H_{20}O_{12}$	18.01	463.09	246.92[a]	515.07[b]
UNPD53212	Hydroxyanthraquinones	$C_{28}H_{36}O_{16}$	18.53	627.19	120.99[a]	234.01[b]
Catechin 7-glucoside	Flavonoid-7-O-glycosides	$C_{21}H_{24}O_{11}$	18.69	451.12	201.61[a]	410.34[b]

Identified compound	Ontology	Molecular Formula	RT	m/z	GRE	β-GRE
Phlorizin	Flavonoid O-glycosides	$C_{21}H_{24}O_{10}$	18.94	435.13	897.19[a]	1918.63[b]
Kaempferol 3-[2''-(p-coumaroylglucosyl)rhamnoside]	Flavonoid-3-O-glycosides	$C_{36}H_{36}O_{17}$	19.12	739.19	319.19[a]	722.94[b]
Plumieride	Iridoid O-glycosides	$C_{21}H_{26}O_{12}$	19.19	469.14	374.21[a]	599.66[b]

RT – retention time in minutes, GRE- aqueous green rooibos extract and β-GRE – betacyclodextrin assisted extracts of green rooibos expressed at mg.kg-1 of catechin.

Table S4: Furfural content of canned apples with added crude green rooibos extracts.

Storage		Samples type and concentration				
**Temp**	**Time**	**Control**	**Gre 0.25**	**Gre 0.5**	**β-Gre 0.25**	**β-Gre 0. 5**
	0	1.75 ± 0.12^{ad}	1.62 ± 0.10^{ad}	2.06 ± 0.27^{af}	1.42 ± 0.36^{ab}	1.54 ± 0.3^{ac}
	4	1.39 ± 0.18^{a}	1.59 ± 0.18^{ad}	2.10 ± 0.48^{af}	1.24 ± 1.11^{a}	1.64 ± 0.14^{ad}
	8	1.28 ± 0.31^{a}	1.68 ± 0.13^{ad}	2.18 ± 0.29^{af}	1.70 ± 0.89^{ad}	1.78 ± 0.21^{ad}
	12	1.92 ± 0.44^{ae}	1.59 ± 0.54^{ad}	2.09 ± 0.78^{af}	1.39 ± 0.42^{a}	1.56 ± 0.42^{ad}
23°C	16	3.47 ± 1.39^{gi}	1.71 ± 0.22^{ad}	2.40 ± 0.70^{cf}	1.77 ± 0.14^{ad}	1.73 ± 0.14^{ad}
	20	3.69 ± 1.24^{hi}	2.08 ± 0.15^{af}	2.76 ± 0.71^{eg}	1.96 ±1.16^{ae}	2.39 ± 0.16^{bf}
	24	4.18 ±0.64^{i}	2.52 ± 0.31^{df}	3.00 ± 0.78^{fh}	2.13 ± 1.26^{af}	2.42 ± 0.26^{cf}
	0	1.75 ± 0.21^{A}	1.62 ± 0.13^{A}	2.06 ± 0.26^{A}	1.42 ± 0.30^{A}	1.54 ± 0.30^{A}
	4	2.81 ± 0.33^{AC}	2.31 ± 0.14^{A}	2.19 ± 0.17^{A}	2.40 ± 0.08^{AB}	2.57 ± 0.25^{AB}
	8	6.62 ± 0.28^{GJ}	4.88 ± 0.60^{DF}	4.10 ± 0.84^{CE}	4.80 ± 0.42^{DF}	3.84 ± 1.19^{BD}
37°C	12	7.47 ± 0.77^{IK}	6.09 ± 0.69^{FI}	5.42 ± 0.45^{EG}	5.68 ± 1.75^{FG}	5.91 ± 0.49^{FH}
	16	12.04 ± 0.55^{M}	7.98 ± 0.48^{JK}	8.68 ± 1.19^{K}	6.43 ± 1.22^{GI}	7.36 ± 0.44^{HK}
	20	17.10 ± 1.26^{O}	14.06 ± 1.02^{N}	10.81 ± 1.20^{LM}	10.37 ± 0.54^{L}	10.60 ± 1.71^{L}
	24	24.92 ± 0.54^{Q}	22.39 ± 0.15^{P}	13.90 ± 1.91^{N}	16.38 ± 0.98^{O}	14.37 ± 0.62^{N}

Data presented as furfural content (mg.100g^{-1}) of canned apples with added green rooibos extracts stored at 23 and 37°C for 24 weeks expressed as mean ± standard deviation (n = 3). ^{abc} Means with different letter superscripts of the same upper or lowercase on the same row denotes significant differences ($p < 0.05$). Gre 0.25 – 0.5 green rooibos native extract at 0.25 and 0.5%, β-GRE 0.25 – 0.5 betacyclodextrin encapsulated green rooibos extract.

Table S5: Hydroxymethyl furfural content of canned apples with added crude green rooibos extracts.

Storage		Samples type and concentration				
**Temp**	**Time**	**Control**	**Gre 0.25**	**Gre 0.5**	**β-Gre 0.25**	**β-Gre 0. 5**
23°C	0	0.65 ± 0.68^{a}	0.62 ± 0.48^{a}	0.71 ±1.1^{a}	0.59 ± 1.88^{a}	0.61 ± 0.92^{a}
	4	0.78 ± 0.62^{ab}	0.64 ± 0.09^{ab}	0.72 ± 0.55^{a}	0.74 ± 0.96^{ab}	0.80 ± 0.19^{ab}
	8	1.63 ± 0.73^{jl}	1.44 ± 1.02^{hi}	1.14 ± 0.29^{cf}	1.09 ± 0.45^{ce}	0.94 ± 0.66^{b}
	12	1.76 ± 0.58^{jl}	1.63 ± 0.55^{jl}	1.29 ± 0.44^{eh}	1.18 ± 0.77^{cf}	1.01 ± 0.17^{cd}
	16	1.95 ± 0.77^{l}	1.78 ± 0.78^{kl}	1.44 ± 0.84^{hi}	1.28 ± 1.14^{eh}	1.22 ± 0.82^{eg}
	20	2.33 ± 1.66^{m}	1.93 ± 1.07^{l}	1.56 ± 1.94^{ij}	1.19 ± 0.87^{df}	1.31 ±1.36^{eh}
	24	2.79 ± 1.11^{n}	2.46 ± 1.62^{m}	1.76 ± 1.02^{jl}	1.31 ± 0.98^{eh}	1.36 ±1.66^{gh}
37°C	0	0.65 ± 1.08^{AB}	0.62 ± 0.46^{A}	0.71 ± 0.86^{AB}	0.59 ± 0.81^{A}	0.61 ± 0.92^{A}
	4	0.92 ± 0.78^{B}	0.76 ± 0.81^{AB}	0.85 ± 0.77^{AB}	0.79 ± 0.89^{AB}	0.84 ± 1.01^{AB}
	8	2.20 ± 0.59^{FG}	1.90 ± 1.14^{DE}	1.47 ± 1.92^{C}	1.88 ± 1.24^{DE}	1.76 ± 0.96^{DE}
	12	2.91 ± 0.14^{J}	1.91 ± 0.98^{DE}	1.71 ±1 .06^{CD}	2.04 ± 0.78^{EG}	1.81 ± 0.94^{DE}
	16	3.36 ± 1.03^{K}	2.28 ± 0.1^{GH}	2.52 ± 1.12^{HI}	1.96 ± 1.40^{DF}	2.04 ± 1.15^{EG}
	20	4.46 ± 0.67^{M}	3.53 ± 0.89^{K}	3.43 ± 0.97^{K}	2.77 ± 2.58^{IJ}	2.79 ± 0.05^{J}
	24	5.67 ± 0.96^{P}	5.09 ± 1.09^{O}	4.76 ± 1.33^{N}	3.86 ± 1.07^{L}	3.48 ± 1.03^{K}

Data presented as hydroxymethyl furfural content (mg.100g^{-1}) of canned apples with added green rooibos extracts stored at 23 and 37°C for 24 weeks expressed as mean ± standard deviation (n = 3). Gre 0.25 – green rooibos native extract at 0.25%, β-GRE 0.25 – betacyclodextrin encapsulated green rooibos extract.
